# Succinate Regulates Endothelial Mitochondrial Function and Barrier Integrity

**DOI:** 10.3390/antiox13121579

**Published:** 2024-12-21

**Authors:** Reham Atallah, Juergen Gindlhuber, Wolfgang Platzer, Rishi Rajesh, Akos Heinemann

**Affiliations:** 1 Otto Loewi Research Center for Vascular Biology, Immunology and Inflammation, Division of Pharmacology, Medical University of Graz, 8010 Graz, Austria; 2 Otto Loewi Research Center for Vascular Biology, Immunology and Inflammation, Division of Physiology & Pathophysiology, Medical University of Graz, 8010 Graz, Austria

**Keywords:** endothelial cells, succinate, mitochondrial function, barrier integrity, reactive oxygen species

## Abstract

Endothelial dysfunction is a hallmark of several pathological conditions, including cancer, cardiovascular disease and inflammatory disorders. In these conditions, perturbed TCA cycle and subsequent succinate accumulation have been reported. The role of succinate as a regulator of immunological responses and inflammation is increasingly being recognized. Nevertheless, how endothelial cell function and phenotype are altered by elevated intracellular succinate has not been addressed yet. Thus, we employed numerous in vitro functional assays using primary HUVECs and diethyl succinate (DES), a cell membrane-permeable succinate analogue. An MTS assay 1 h post stimulation with DES suggested reduced metabolic activity in HUVECs. Concurrently, elevated production of ROS, including mitochondrial superoxide, and a reduction in mitochondrial membrane potential were observed. These findings were corroborated by Seahorse mito-stress testing, which revealed that DES acutely lowered the OCR, maximal respiration and ATP production. Given the link between mitochondrial stress and apoptosis, we examined important survival signalling pathways. DES transiently reduced ERK1/2 phosphorylation, a response that was followed by a skewed pro-apoptotic shift in the BAX to BCL2L1 gene expression ratio, which coincided with upregulating VEGF gene expression. This indicated an induction of mixed pro-apoptotic and pro-survival signals in the cell. However, the BAX/BCL-XL protein ratio was unchanged, suggesting that the cells did not commit themselves to apoptosis. An MTS assay, caspase 3/7 activity assay and annexin V/propidium iodide staining confirmed this finding. By contrast, stimulation with DES induced acute endothelial barrier permeability, forming intercellular gaps, altering cell size and associated actin filaments without affecting cell count. Notably, during overnight DES exposure gradual recovery of the endothelial barrier and cell sprouting was observed, alongside mitochondrial membrane potential restoration, albeit with sustained ROS production. COX-2 inhibition and EP4 receptor blockade hindered barrier restoration, implicating a role of COX-2/PGE_2_/EP4 signalling in this process. Interestingly, ascorbic acid pre-treatment prevented DES-induced acute barrier disruption independently from ROS modulation. In conclusion, succinate acts as a significant regulator of endothelial mitochondrial function and barrier integrity, a response that is counterbalanced by upregulated VEGF and prostaglandin production by the endothelial cells.

## 1. Introduction

The endothelium is a monolayer of cells lining blood vessels that forms a barrier between the blood and surrounding tissues. With this localization, endothelial cells also regulate vascular tone, mediate immune cell extravasation, prevent thrombosis and support the growth and development of connective tissue cells [1,2]. Recently, a role of metabolites in regulating endothelial cell activation and function has been described [3]. For instance, lactate induces vascular endothelial permeability via the disruption of VE-cadherin in a G-protein coupled receptor 81 (GPR81)-dependent manner [4]. In parallel, increasing evidence points towards metabolic adaptations in endothelial cells in response to constant changes in the microenvironment. This metabolic rewiring is becoming more acknowledged as a mandate to maintain endothelial homeostasis and activation [5]. As an example, subsequent to activation of the transcription factor forkhead box O1 (FOXO1), a major driver of endothelial cell quiescence, the metabolite S-2 hydroxyglutarate is produced, limiting cell cycle progression, metabolic activity and vascular expansion [6]. In contrast, in response to stimulation with vascular endothelial growth factor (VEGF), endothelial cells upregulate glycolysis, a process that is required for endothelial sprouting and vessel outgrowth, and silencing of the glycolytic regulator 6-phosphofructo-2-kinase/fructose-2,6-biphosphatase 3 enzyme (PFKFB3) hampers angiogenesis [7]. Therefore, in-depth investigation of the interplay between endothelial cells and metabolites is critical for understanding the pathophysiology of diseases associated with endothelial dysfunction, such as cancer, cardiovascular disorders and inflammatory diseases [8,9,10], and for identifying novel targets aiming to restore endothelial and tissue function.

Succinate is an essential metabolite in the tricarboxylic acid (TCA) cycle. The localization and concentration of this metabolite under physiological conditions is tightly controlled [11]. Under stress conditions, disruption of the TCA cycle may result in the elevation of succinate in the mitochondrial matrix and in the cytosol [12]. Intracellular accumulation of succinate can also occur via uptake from the extracellular environment [13,14]. In the mitochondrial matrix, succinate accumulation contributes reducing power to the respiratory chain and thus regulates the production of reactive oxygen species (ROS) [15], while cytosolic succinate is reported to inhibit α-ketoglutarate-dependent dioxygenases, a family of enzymes involved in a wide range of biological processes, including regulation of hypoxia-inducible factor (HIF), formation of extracellular matrix, epigenetic regulation of gene transcription and rewiring of cellular metabolism [16]. Elevation of intracellular succinate has been reported in several cell types, including endothelial cells, which accumulate succinate intracellularly in response to hypoxia and proinflammatory stimuli such as lipopolysaccharide (LPS) increasing their migratory and invasive properties [17]. Likewise, LPS-stimulated macrophages accumulate succinate, which leads to increased interleukin-1β (IL-1β) expression [18]. Furthermore, loss of the tumour suppressor phosphatase and tensin-homolog (PTEN) in human prostate cancer cells is associated with elevated intracellular succinate levels and increased succinate-supported respiration, a metabolic shift confirming the importance of succinate metabolism in cancer cells [19].

In addition to the regulatory functions of intracellular succinate, modulation of many patho-physiological processes has been linked to succinate secretion and ligation to succinate receptor 1 (SUCNR1) on different cell types [20]. Indeed, the responses mediated by intracellular succinate accumulation might be distinct from the responses induced by succinate extracellularly. For instance, in primary microglia treatment with diethyl succinate, DES (a cell-permeable succinate analogue) prevents their conversion into the proinflammatory M1 phenotype induced by LPS, hence playing a protective role [21]. On the other hand, extracellular succinate, via SUCNR1, induces IL-1β production from macrophages resulting in the exacerbation of inflammation [22]. Our previous study demonstrated that extracellular succinate induces endothelial proliferation, migration and sprouting in a SUCNR1-dependent manner [23]. In the current study, we identify key endothelial functions regulated by intracellular succinate elevation reflected by alteration in mitochondrial function and barrier permeability, effects that are partially counterbalanced by prostaglandin and VEGF secretion, leading to partial restoration of cellular barrier, sprouting and recovery of mitochondrial membrane potential.

## 2. Materials and Methods

Chemicals were obtained from Sigma Aldrich (St. Louis, MO, USA), unless specified otherwise.

### 2.1. Cell Culture

Primary pooled human umbilical vein endothelial cells (HUVECs) were purchased from Lonza (Basel, Switzerland). Cells were cultured in Endothelial Cell Growth Medium-2 (EGM-2) Bulletkit (Lonza) in T75 Corning CellBind flasks (Corning, NY, USA) pre-coated with 1% gelatine solution at 21% O_2_, 5% CO_2_ and 37 °C in a 90% humidified incubator. Cells were passaged when 80–90% confluence was reached and detached with trypsin/EDTA solution (Lonza), followed by trypsin neutralization using trypsin-neutralizing solution (Lonza). After a centrifugation step at 220 g for 5 min at RT, cells were seeded for different assays. Cells from passages 3 to 7 were used for experiments.

### 2.2. Electric Impedance Cell–Substrate Sensing (ECIS)

HUVECs were seeded at a density of 40,000 cells/200 µL of complete medium/well of 96W20idf PET arrays (Applied Biophysics, Troy, NY, USA). The wells of the arrays were pre-coated with 10 mM L-cysteine in sterile water, followed by coating with 1% gelatine solution. HUVECs were grown until confluence for ~2 days. On the day of the experiment, cells were serum-starved for 1 h in EGM-2 basal medium supplemented with 2% fetal calf serum (FCS, ThermoFisher Scientific, Waltham, MA, USA), followed by baseline measurement. An ECIS^®^ Z-Theta device (Applied Biophysics) was used to monitor the resistance of the cellular monolayer. Each treatment was performed in triplicates and resistance for each well was recorded at a regular interval at a frequency of 4000 Hz. For certain experiments, U0126 (Cell signaling, Danvers, MA, USA) treatment was performed 30 min before stimulation with DES. Diclofenac treatment was performed 1 h before the addition of DES, while prostaglandin E2 (PGE_2_) receptor (EP2 and EP4) antagonists PF-04418948 (MedChemExpress, Monmouth Junction, NJ, USA) and ONO-AE3-208 (Cayman Chemical, Ann Arbor, MI, USA) were added 2 h post DES. To evaluate the protective capacity of ascorbic acid, cells were treated with ascorbic acid (Lonza) at indicated concentrations for 15 min prior to or simultaneously with DES.

### 2.3. Immunofluorescence

Cells were seeded on gelatine-coated coverslips and grown to confluence before treatment with vehicle or DES in starvation medium for selected time points. At the end of treatment, cells were fixed using 3.7% formalin solution (CarlRoth, Karlsruhe, Germany) for 15 min at RT. The cell monolayer was gently washed three times with phosphate-buffered saline (PBS) and cells were subsequently permeabilized with 0.1% Triton-X-100 in PBS for 10 min. Non-specific binding was blocked by incubation with 10% normal goat serum and 3% bovine serum albumin (BSA) in PBS for 30 min. Next, cells were stained overnight with primary anti-human VE-cadherin antibody (#sc-9989, Santa Cruz, Dallas, TX, USA). In the last step, cells were incubated with a conjugated secondary antibody and conjugated phalloidin (ThermoFisher Scientific) for 30 min. Antifade Mounting Medium with DAPI (Vector laboratories, Newark, CA, USA) was used for counterstaining. Images were acquired using an Olympus (EVIDENT Europe, Hamburg, Germany) VS200 equipped with an Orca-Fusion (Hamamatsu Photonics, Hamamatsu, Japan) camera and 40× UPLXAPO objective (EVIDENT Europe). All conditions were imaged as z-stacks and automatically saved as maximum intensity projections. For each condition, 9 areas of 1 mm^2^ were taken for analysis in Fiji Software (ImageJ v1.53q) [24]. Image analysis consisted of a nucleus quantification and determination of the cell area based on the combined fluorescent signal of VE-cadherin and phalloidin. The generated region of interest was used as a mask for phalloidin intensity measurement and inverted to quantify gaps in the monolayer.

### 2.4. In Vitro Permeability Assay

HUVECs (100,000 cells) were cultured on Transwell inserts (0.4 μm pore size, Corning) of a 24-well plate until confluent. The serum-starved endothelial monolayer was stimulated with medium containing vehicle or 10 mM DES for indicated time points. At the end of incubation, 100 μL of PBS containing FITC-dextran 4000 (MedChemExpress, 50 μg/mL) was added to the upper chamber and 500 μL of PBS was added to the lower chamber. The 24-well plate was incubated for 15 min at 37 °C, and the concentration of FITC-dextran in the lower chamber was determined using a plate reader (BMG Labtech, Ortenberg, Germany) with excitation and emission wavelengths of 492 and 520 nm, respectively. Thrombin was used as a positive control.

### 2.5. MTS Assay

HUVECs were seeded at a density of 20,000 cells/100 µL of complete medium/well in 96-well plates. On the next day, medium was replaced with starvation medium and treatments were added for selected time points. A CellTiter 96^®^ AQueous One Solution Cell Proliferation Assay (MTS, Promega, Madison, WI, USA) was used, according to the manufacturer’s instructions. Absorbance at 490 nm was recorded on a plate reader (Bio-Rad, Hercules, CA, USA).

### 2.6. Cellular ROS Measurement

HUVECs were seeded at a density of 50,000 cells/mL of complete medium/well in 24-well plates. When the monolayer was confluent, cells were changed to starvation medium and treated with either vehicle or DES for selected time points. Dihydrorhodamine 123 (DHR 123, 10 µM) was added for 30 min at 37 °C protected from light. To stop the reaction, samples were transferred to ice, detached and resuspended in Hanks’ Balanced Salt Solution (HBSS) supplemented with 0.1% BSA and were immediately measured by flow cytometry using FACSCanto II (BD Biosciences, Franklin Lakes, NJ, USA) and FlowJo Software (v10.8.1, BD Biosciences) for analysis. Hydrogen peroxide was used as a positive control.

### 2.7. Mitochondrial Superoxide Measurement

Cells were seeded as mentioned previously and treated with vehicle or DES. A MitoSOX Mitochondrial Superoxide Indicator (ThermoFisher Scientific, 5 µM) was used as per the provider’s instructions and the fluorescent signal was measured on BD FACSCanto II. FlowJo Software was used for analysis.

### 2.8. Mitochondrial Membrane Potential Measurement

Cells were seeded on 24-well cell culture plates at a density of 50,000 cells/mL of complete medium/well. Once confluent, cells were treated with vehicle or DES in starvation medium for selected times and a TMRE-Mitochondrial Membrane Potential Assay Kit (Abcam, Cambridge, UK) was used, according to the user manual. The fluorescent signal was recorded using BD FACSCanto II followed by FlowJo Software for analysis. For live cell imaging, cells were seeded on glass-bottom Petri dishes (ThermoFisher Scientific) overnight and subsequently treated with vehicle or DES in starvation medium. Images were acquired on a Nikon-Ti2-E microscope equipped with an Andor Zyla 4.2 PLUS sCMOS camera (Nikon, Tokyo, Japan) and stage incubation system (Okolab, Ottavian, Italy) with a 60× objective and processed in Fiji. Carbonyl cyanide-p-trifluoromethoxyphenylhydrazone (FCCP) was used as a positive control.

### 2.9. Mito-Stress Test

Real-time mitochondrial function assessment was performed using a Seahorse XF Cell Mito Stress Test Kit (#103015-100) and a Seahorse XF Pro Analyzer (Agilent, Santa Clara, CA, USA). In summary, 20,000 cells were seeded in 96-well Seahorse assay plates and allowed to settle overnight. Prior to the assay, cells were washed twice in Agilent Seahorse XF DMEM assay medium (pH 7.4) followed by incubation in the same medium at 37 °C in the absence of carbon dioxide for 1 h. Metabolic assessment was carried out as per the manufacturer’s protocol. The real-time oxygen consumption rate (OCR) and extracellular acidification rate (ECAR) were measured in response to subsequent injections of DES at selected concentrations, 2.5 µM oligomycin, 1 µM FCCP and 0.5 µM mixture of rotenone and antimycin.

### 2.10. Caspase 3/7 Assay

The activity of caspase 3/7 was measured using a Caspase-Glo^®^ 3/7 Assay System (Promega), according to the manufacturer’s instructions.

### 2.11. Western Blotting

After washing with HBSS, cells were lysed in RIPA buffer supplemented with 2X protease/phosphatase inhibitor cocktail (100X Halt Protease and Phosphatase inhibitor cocktail, ThermoFisher Scientific) via sonication (4 cycles at 40% power for 10 s each) followed by centrifugation at 12,000 rpm for 10 min at 4 °C. The supernatant containing protein extracts was used for Western blotting. Total protein concentration was determined using a Pierce™ BCA Protein Assay Kit (ThermoFisher Scientific), according to the manufacturer’s instructions, and ~20 µg of protein was loaded onto a precast gradient gel (Novex WedgeWell Tris-Glycine gels, 4–20%, XP04205, ThermoFisher Scientific). The gel was run at 200 V for ~40 min. Protein bands were transferred onto a PVDF membrane using an iBlot™ 2 Dry Blotting System (Thermofisher Scientific). Unspecific binding was blocked with 5% skimmed milk in Tris-Buffered Saline with Tween (TBST) buffer on a shaker at RT for 1 h. For phosphorylated and total extracellular regulated kinase (ERK1/2), the membrane was blocked in 5% BSA in TBST. Membranes were incubated with primary antibody (phosphorylated ERK #4370, total ERK #4695, Cell signaling), according to the manufacturer’s indicated dilution and diluent at 4 °C overnight. BCL-2 Associated X (BAX) antibody was kindly provided by Prof. Michael Dengler at the Medical University of Graz [25], while B-cell lymphoma-extra large (BCL-XL) antibody was purchased from cell signaling (#2764). Anti-cyclooxygenase-2 (COX-2) antibody was purchased from Abcam (#Ab15191). The next day, membranes were incubated with respective horseradish peroxidase-conjugated secondary antibodies (Jackson Immunoresearch, West Grove, PA, USA) for 1.5 h. After washing, bands were visualized by incubation for 5 min with Clarity Western ECL Blotting Substrate (Bio-Rad) and subsequently evaluated with a Bio-Rad chemiluminescence detector and corresponding Software (ImageLab v6.0.1). As required, membranes were stripped for 30 min with stripping buffer (ThermoFisher Scientific) at RT with shaking, washed three times with TBST buffer for 10 min, blocked for 60 min at RT and incubated with subsequent primary antibody and corresponding secondary antibody on the following day.

### 2.12. Real-Time PCR

Cells were harvested in TRIzol™ Reagent (ThermoFisher Scientific) and mRNA was extracted as per the product’s manual. A total of 1 μg of RNA was reverse-transcribed using a High-Capacity cDNA Reverse Transcription Kit (Thermofisher Scientific), according to the manufacturer’s instructions. SsoAdvanced™ Universal SYBR^®^ Green Supermix (Bio-Rad) and PrimePCR™ SYBR^®^ Green Assay primers (Bio-Rad) were used (BAX: qHsaCED0037943; BCL2L1: qHsaCED0036793; VEGFA: qHsaCED0006937). Samples were run in duplicates and target genes were normalized to β-actin Cq values.

### 2.13. Radioimmunoassay

Radioimmunoassay for the quantification of PGE_2_ was performed as previously described [26].

### 2.14. Spheroid Sprouting Assay

Cells were resuspended in 0.3% methylcellulose solution in M199 (Lonza) supplemented with serum and penicillin/streptomycin (Thermofisher Scientific). Spheroids were generated by overnight incubation in a hanging position at 37 °C. 1X HBSS with 10% FCS was used to harvest the spheroids. The spheroid suspension was centrifuged for 5 min at 300 g at RT without brake. The pellet was overlaid with 1.2% methylcellulose stock solution containing 40% FCS, NaHCO_3_ (15.6 mg/mL), type-1 collagen (2 mg/mL, Corning) and NaOH (1 M) on ice following this order. Collagen-spheroid solution was pipetted in a 24-well plate and incubated at 37 °C for 2 h to allow collagen to polymerize. Stimulation media containing either vehicle or DES was added. Spheroids were stimulated for 16 h before termination using 3.7% formalin solution. Positive control for this assay was composed of basic fibroblast growth factor (b-FGF, 10 ng/mL, Reliatech, Wolfenbüttel, Germany) + VEGF (25 ng/mL, ReliaTech) + tumour necrosis factor alpha (TNFα, 10 ng/mL, ReliaTech). Automated z-stack images were generated on a Nikon-Ti2-E microscope using a 10× objective and sprouting parameters were calculated using NIS Elements (v5.21.03, Nikon) GA3 Software.

### 2.15. Annexin V/Propidium Iodide Staining

Serum-starved HUVECs were incubated with either vehicle or succinate for 16 h. The cells were detached using trypsin/EDTA followed by trypsin inactivation using trypsin-neutralizing solution. After a centrifugation step at 220 g for 5 min, cells were resuspended in a cocktail of annexin V (Biolegend, San Diego, CA, USA) and propidium iodide (Thermo Fisher Scientific) for 15 min at RT in the dark. Analysis was performed using BD FACSCanto II and FlowJo Software. Staurosporine was used as a positive control.

### 2.16. Statistical Analysis

Data are presented as mean and SEM for n observations, where n denotes independent experiments, performed in technical duplicates or triplicates. Comparisons among groups were performed using *t*-tests, or 1-way or 2-way ANOVA for repeated measures followed by Tukey’s post hoc test based on the number of variables, using GraphPad Prism (v9.5.1, GraphPad Software, La Jolla, CA, USA). Probability values of *p* < 0.05 were considered statistically significant.

## 3. Results

### 3.1. Mitochondrial Function of HUVECs Is Altered in Response to Treatment with DES

**Figure 1:**
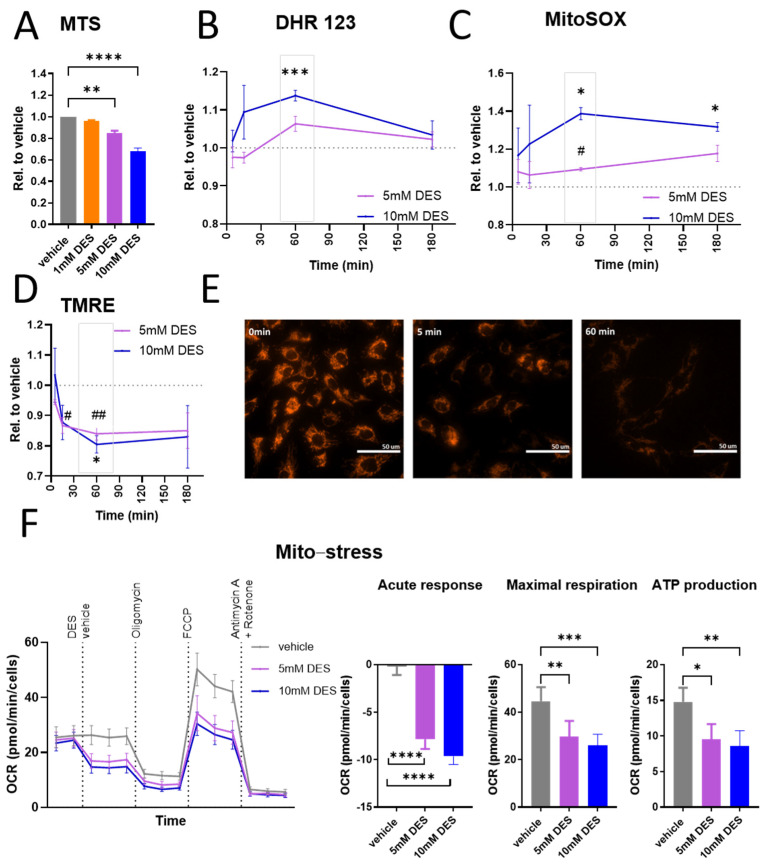
Effect of DES on metabolic and mitochondrial function in HUVECs. (**A**) MTS assay of HUVECs stimulated with DES at indicated concentrations for 1 h (n = 3). (**B**) DHR 123 geometric mean of fluorescence intensity in HUVECs stimulated with DES at indicated concentrations for selected time points (n = 5). (**C**) MitoSOX geometric mean of fluorescence intensity in HUVECs stimulated with DES at indicated concentrations for selected time points (n = 3). (**D**) TMRE geometric mean of fluorescence intensity in HUVECs stimulated with DES at indicated concentrations for selected time points (n = 4). For (**B**–**D**), the dotted line refers to vehicle-treated cells. (**E**) Representative microscopic image for 3 independent experiments with TMRE in HUVECs stimulated with 10 mM DES for indicated times. (**F**) Mito-stress assay of HUVECs demonstrating OCR with sequential addition of treatments. Acute response to DES, maximal respiration and ATP production were calculated (n = 5). For (**A**–**D**,**F**), data are presented as mean and SEM, with statistical significance determined using two-way ANOVA for repeated measures followed by Tukey’s post hoc test (**B**–**D**) or one-way ANOVA for repeated measures followed by Tukey’s post hoc test (**A**,**F**). For (**C**,**D**), * refers to comparison between vehicle and 10 mM DES, while # refers to comparison between vehicle and 5 mM DES. *, # *p* < 0.05; **, ## *p* < 0.01; *** *p* < 0.001; **** *p* < 0.0001.

Since succinate accumulation is a hallmark of many pathological conditions, such as inflammatory disorders, cardiovascular diseases and cancer [17,27,28,29], we sought to investigate how endothelial cell function is altered by increasing succinate concentrations intracellularly. Stimulation of primary HUVECs with DES in the micromolar range (1–500 μM) did not induce any significant changes in cellular metabolic activity measured in an MTS assay at 16 h post stimulation or in cellular barrier function monitored over 16 h by ECIS (Supplementary Figure S1A,B). Therefore, succinate concentrations in the millimolar range (1–10 mM), corresponding to reported pathological succinate concentrations [27,28], were used for all assays. Since DES is known to be taken up and metabolized in the TCA cycle in the mitochondria [30], and given the role of succinate as a messenger linking mitochondrial function to important cellular responses such as angiogenesis, metastasis and inflammation [31,32], we sought to perform a comprehensive assessment of the mitochondrial function of HUVECs upon simulation with DES. The MTS assay performed at 1 h post treatment revealed a decrease in the overall metabolic activity of cells treated with 5 mM or 10 mM DES (Figure 1A). Further, in DHR 123-labelled cells, DES caused a gradual increase in ROS production that reached a statistically significant peak at 1 h post treatment with 10 mM DES (Figure 1B). Similarly, an increase in mitoSOX fluorescence was observed with 5 mM DES 1 h post treatment and with 10 mM at 1 h and 3 h post treatment (Figure 1C), indicating an elevation in superoxide anion production in the mitochondria. These data together suggest that DES triggers ROS production in HUVECs, which originates at least partly in the mitochondria. We also investigated the mitochondrial membrane potential using TMRE. Interestingly, a gradual reduction was observed and was statistically significant at 30 min and 1 h post treatment with 5 mM DES and 1 h post treatment with 10 mM DES (Figure 1D). These results were visualized by live cell microscopy of HUVECs treated with 10 mM DES at 5 min and 1 h (Figure 1E). Since the mitochondrial membrane potential is maintained by complexes I, III and IV, we hypothesize that high succinate concentrations in complex II cause impaired electron transport and, thus, reduced proton gradient maintenance, which is required for ATP production. Indeed, and in line with the reduced mitochondrial membrane potential, an acute reduction in OCR associated with reduced ATP production and reduced maximal respiration were measured in a Seahorse mito-stress test upon treatment with DES (Figure 1F). In contrast, there were no significant changes in ECAR (Supplementary Figure S2), indicating that HUVECs, which are known to rely largely on glycolysis to meet their metabolic needs [33], do not further upregulate this metabolic pathway to compensate for reduced mitochondrial function. Collectively, these data suggest that DES causes an acute alteration in HUVECs mitochondrial function, presenting as a reduction in the mitochondrial membrane potential, OCR, maximal respiration and ATP production, while sustaining increased ROS production.

### 3.2. DES Does Not Induce Apoptosis in HUVECs

**Figure 2:**
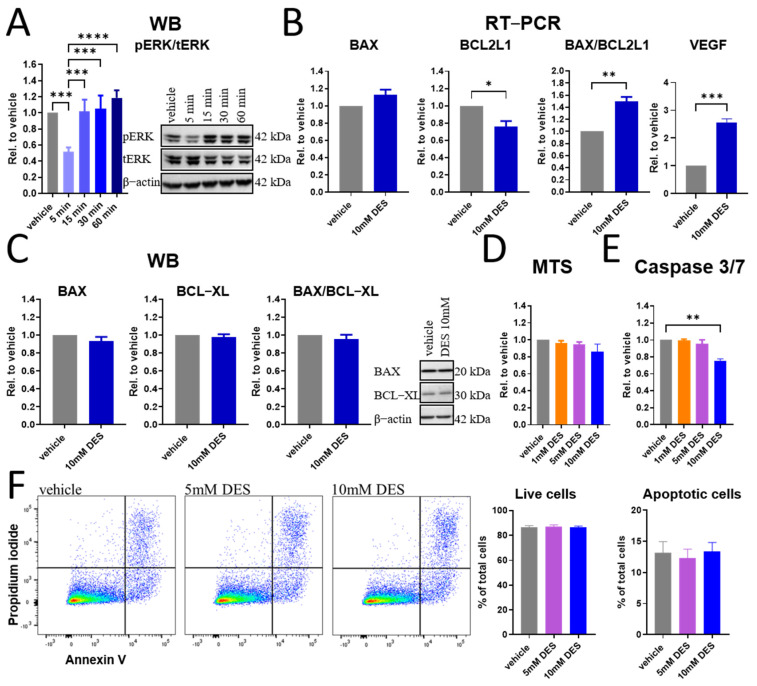
Effect of DES on HUVECs viability. (**A**) Western blot of phosphorylated ERK1/2 and ratio of pERK/tERK/β-actin in HUVECs stimulated with 10 mM DES for indicated time points (n = 4). (**B**) RT-PCR of BAX, BCL2L1 and VEGF mRNA expression in HUVECs stimulated with 10 mM DES for 4 h (n = 5). (**C**) Western blot of BAX and BCL-XL in HUVECs stimulated with 10 mM DES for 6 h (n = 5). (**D**) MTS assay of HUVECs stimulated with indicated DES concentrations for 6 h (n = 3). (**E**) Caspase 3/7 activity of HUVECs post treatment with indicated DES concentrations for 16 h (n = 3). (**F**) Representative dot plot of annexin V/propidium iodide staining of HUVECs treated with indicated DES concentrations for 16 h. Live and apoptotic cells were quantified (n = 9). Data are presented as mean and SEM, with statistical significance determined using one-way ANOVA for repeated measures followed by Tukey’s post hoc test (**A**,**D**–**F**) or paired *t*-test (**B**,**C**). * *p* < 0.05; ** *p* < 0.01; *** *p* < 0.001; **** *p* < 0.0001.

As mitochondrial stress could eventually culminate in cellular death and apoptosis, we investigated hallmarks linked to cell viability and survival. We first investigated the ERK pathway, which is a critical signalling cascade involved in cell survival and function [34], and we observed an acute reduction in phosphorylated ERK1/2 levels 5 min post treatment with 10 mM DES, suggesting that DES initially inhibits this important pathway. Nevertheless, this reduction was transient and the cells quickly restored normal ERK activity (Figure 2A).

We further investigated the gene expression of pro-apoptotic BAX and anti-apoptotic BCL2-like 1 (BCL2L1) signals at 4 h post stimulation with 10 mM DES, where the ratio of both suggested priming of the cells towards an apoptotic process. Interestingly, at the same time, upregulation of VEGF gene expression was detected, which is a major growth factor involved in cell survival, angiogenesis and barrier permeability [35] (Figure 2B). This increase demonstrated possible induction of compensatory mechanisms inside the cells to promote their survival in response to DES-induced stress. At 6 h post stimulation, there were no detectable changes in the protein products of BAX and BCL2L1 (BAX and BCL-XL), suggesting that the initial gene alterations did not result in changes in protein production (Figure 2C). At this time point, the MTS assay demonstrated no significant reduction in response to DES, indicating probable restoration of cellular metabolic activity (Figure 2D). We moved further to look into the activity of key executor caspases in the cells (caspase 3/7) at 16 h post treatment, where a reduction was detected (Figure 2E), demonstrating that despite the early pro-apoptotic signal, the cells did not proceed in apoptosis. These data were further corroborated in annexin V/propidium iodide staining, where no significant changes in live or apoptotic cell percentages were determined (Figure 2F). Altogether, these data demonstrate a complex time-dependent response of HUVECs to DES stimulation involving early induction of pro-apoptotic and pro-survival signals that seemingly override the pro-apoptotic signals leading to cellular survival.

### 3.3. DES Induces an Acute Disruption in HUVECs Barrier

**Figure 3:**
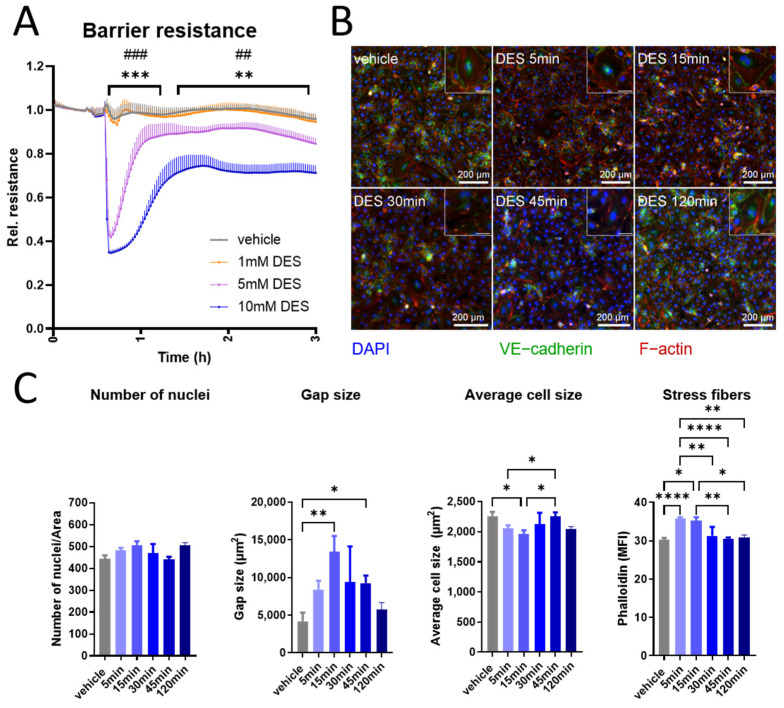
Acute effect of DES on HUVECs barrier integrity. (**A**) Resistance of HUVECs monolayer stimulated with DES at indicated concentrations (n = 4). (**B**) Immunofluorescence staining of VE-cadherin and F-actin in HUVECs stimulated with 10 mM DES at selected time points. The image is representative of 3 independent experiments. Zoomed-in images are shown on the upper right side for each condition, scale bar = 50 µm. (**C**) Image analysis demonstrating number of nuclei, gap size, average cell size and median fluorescence intensity (MFI) of phalloidin-stained stress fibres. Data are presented as mean and SEM, with statistical significance determined using two-way ANOVA for repeated measures followed by Tukey’s post hoc test (**A**) or one-way ANOVA for repeated measures followed by Tukey’s post hoc test (**C**). For (**A**), * refers to comparison between vehicle and 10 mM DES, while # refers to comparison between vehicle and 5 mM DES. * *p* < 0.05; **, ## *p* < 0.01; ***, ### *p* < 0.001; **** *p* < 0.0001.

Next, we investigated the electrical impedance of the endothelial monolayer upon stimulation with DES, where 5 to 10 mM DES caused an acute concentration-dependent reduction. This decline was maximal within minutes post treatment and was reversed in the next 1–2 h, but still remained under baseline (Figure 3A). These results were corroborated in an FITC-dextran in vitro permeability assay, which demonstrated a transient increase in the monolayer permeability in response to stimulation with 10 mM DES (Supplementary Figure S3). In line with these findings, immunofluorescence staining for VE-cadherin and F-actin (a representative image is shown in Figure 3B) revealed that treatment with 10 mM DES caused no significant reduction in the number of nuclei, confirming no direct toxic effect of the treatment. On the contrary, gap size in the cell monolayer increased to reach a maximum at 15 min post treatment, after which it gradually returned towards baseline. Gap formation was accompanied by cell contraction, as reflected in reduced cell sizes, with subsequent cell spreading suggesting cytoskeletal rearrangement. The intensity of F-actin staining, which supports contractile force to the cells [36], correlated with the changes in cell size (Figure 3C). Collectively, these data demonstrate that DES acutely disrupts the endothelial barrier with the formation of intercellular gaps and cytoskeletal reorganization.

### 3.4. Gradual Restoration of HUVECs Barrier and Sprouting During Overnight Stimulation with DES

**Figure 4:**
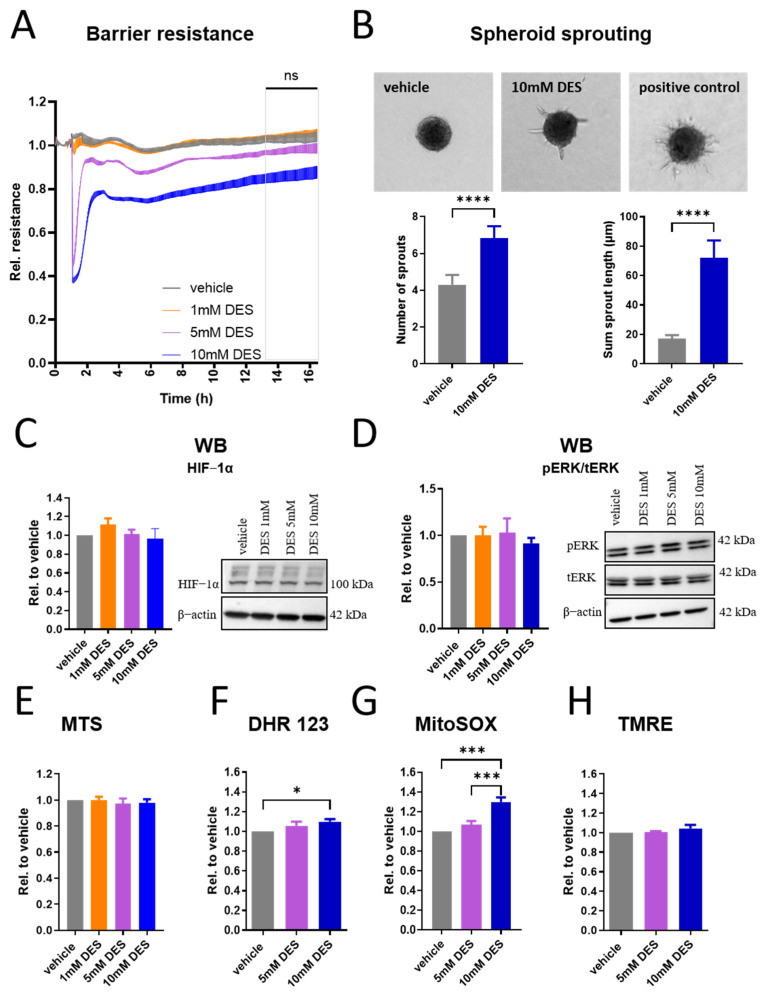
Restoration of HUVECs barrier and sprouting during overnight stimulation with DES. (**A**) Resistance of HUVECs monolayer with overnight stimulation with DES at indicated concentrations (n = 6). (**B**) Spheroid sprouting assay of HUVECs stimulated with 10 mM DES for 16 h. Number of sprouts and total sprout length were calculated. The image is representative of three independent experiments. (**C**) Western blot of HIF-1α in HUVECs stimulated with DES at indicated concentrations for 16 h (n = 8). (**D**) Western blot of phosphorylated ERK1/2 and ratio of pERK/tERK/β-actin in HUVECs stimulated with indicated DES concentrations for 16 h (n = 4). (**E**) MTS assay of HUVECs stimulated with DES at indicated concentrations for 16 h (n = 3). (**F**) DHR 123 geometric mean of fluorescence intensity in HUVECs stimulated with DES at indicated concentrations for 16 h (n = 6). (**G**) MitoSOX geometric mean of fluorescence intensity in HUVECs stimulated with DES at indicated concentrations for 16 h (n = 5). (**H**) TMRE geometric mean of fluorescence intensity in HUVECs stimulated with DES at indicated concentrations for 16 h (n = 4). Data are presented as mean and SEM, with statistical significance determined using two-way ANOVA for repeated measures followed by Tukey’s post hoc test (**A**), paired *t*-test (**B**) or one-way ANOVA for repeated measures followed by Tukey’s post hoc test (**C**–**H**). ns refers to no statistical difference between vehicle and either 5 mM DES or 10 mM DES. * *p* < 0.05; *** *p* < 0.001; **** *p* < 0.0001.

Since the acute responses of HUVECs to DES stimulation were significant, we next assessed their response to prolonged stimulation. During overnight exposure, HUVECs appeared to gradually restore their barrier integrity, suggesting cellular adaptation following the initial disruption (Figure 4A). In line with this, cell sprouting in a spheroid assay, measured as sprout number and length, confirmed the induction of cell migration, which is a key angiogenic function often engaged after changes in barrier permeability (Figure 4B). Parallel increase in total VE-cadherin protein levels (Supplementary Figure S4A) suggested that the cells increase the synthesis of proteins crucial for re-establishing adherens junctions, likely supporting barrier restoration after initial disruption. To explore possible mechanisms underlying observed cellular adaptation, we examined HIF-1α expression, given the known role of succinate in stabilizing this factor [31]. Although there was a trend towards increased HIF-1α mRNA expression in response to DES (Supplementary Figure S4B), no significant change at the protein level was observed (Figure 4C). Further analysis of the ERK pathway revealed no additional significant changes, indicating that this pathway is no longer by altered treatment (Figure 4D). Since ERK1/2 was acutely downregulated post stimulation with DES and we had no indication of cellular apoptosis, we investigated a possible role of ERK1/2 in DES-induced barrier response. Therefore, we pre-treated the cells with 10 µM U0126, a MEK1/2 inhibitor upstream of ERK1/2 [37], followed by 5–10 mM DES stimulation, and monitored the resistance of the monolayer using ECIS. Our results showed that U0126 pre-treatment enhanced the speed of barrier recovery following acute DES-induced disruption, as indicated by an accelerated return of electrical impedance close to baseline. However, this response was not sustained as prolonged inhibition resulted in the deterioration of barrier integrity (Supplementary Figure S4C). These findings indicate that ERK1/2 inhibition at earlier stages of barrier disruption facilitates rapid recovery, whereas sustained ERK inhibition has a detrimental effect on long-term barrier stabilization and recovery. Next, we investigated the metabolic and mitochondrial functions that were initially affected by the treatment, where the overall metabolic activity of the cells in the MTS assay was not different in response to DES (Figure 4E). Interestingly, ROS production was still sustained in DHR 123 and MitoSOX assays (Figure 4F,G), while the mitochondrial membrane potential was restored (Figure 4H). Taken together, these data indicate that the cells, at this point, are able to restore their barrier integrity and show a migratory phenotype, and have successfully recovered important aspects of mitochondrial function.

### 3.5. COX-2/PGE_2_/EP4 Signalling Axis Plays a Role in the Recovery of HUVECs Barrier

**Figure 5:**
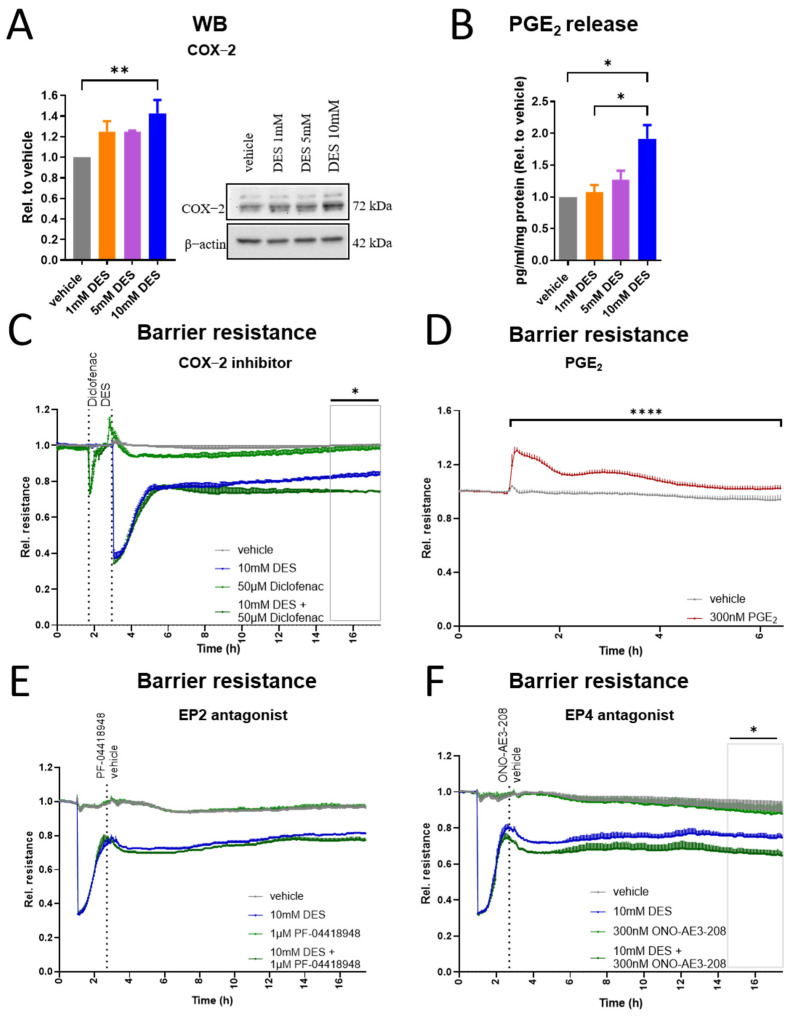
COX-2/PGE_2_/EP4 contribution to barrier recovery in HUVECs. (**A**) Western blot of COX-2 in HUVECs after 16 h of stimulation with DES at indicated concentrations (n = 6). (**B**) Radioimmunoassay for quantification of PGE_2_ in supernatants of HUVECs stimulated with DES for 16 h (n = 3). (**C**) Resistance of HUVECs monolayer treated with diclofenac ~1 h prior to stimulation with 10 mM DES (n = 3). (**D**) Resistance of HUVECs monolayer stimulated with PGE_2_ (n = 3). (**E**) Resistance of HUVECs monolayer treated with EP2 antagonist ~2 h post treatment with 10 mM DES (n = 3). (**F**) Resistance of HUVECs monolayer treated with EP4 antagonist ~2 h post treatment with 10 mM DES (n = 3). Data are presented as mean and SEM, with statistical significance determined using one-way ANOVA for repeated measures followed by Tukey’s post hoc test (**A**,**B**) or two-way ANOVA for repeated measures followed by Tukey’s post hoc test (**C**–**F**). * *p* < 0.05; ** *p* < 0.01; **** *p* < 0.0001.

Given that alterations in the endothelial barrier are usually associated with inflammation and COX-2 is known to be a very important modulator of endothelial response during an inflammatory process [38], we investigated its expression in HUVECs after overnight treatment with DES and observed increased COX-2 protein expression (Figure 5A). Concordantly, the level of PGE_2_, an important modulator of vascular barrier integrity [39], was increased in the cell supernatants (Figure 5B). To address the relevance of this increase regarding the barrier function of the cells, we treated the cells with a COX-1/2 inhibitor, diclofenac (50 µM) [40], 1 h before stimulation with DES. Our results show that COX-2 inhibition partially compromised the barrier recovery of the cells (Figure 5C), indicating that COX-2 is critical for the restoration of the monolayer resistance. To investigate whether the observed PGE_2_ increase contributes to this cellular function, we treated the cells with PGE_2_ and recorded their resistance. Indeed, PGE_2_ enhanced the cellular barrier function (Figure 5D). Since PGE_2_ induces its responses through specific G-protein-coupled prostanoid receptors, among which EP2 and EP4 are expressed by HUVECs [41,42], we blocked these receptors in further experiments using selective antagonists. PF-04418948 (1 µM) or ONO-AE3-208 (300 nM) were added past the acute phase of barrier drop in response to DES. Only ONO-AE3-208, but not PF-04418948, compromised the following recovery of the endothelial barrier (Figure 5E,F), demonstrating that EP4, but not EP2, receptors are involved in the restoration of the cellular barrier. Taken together, these results suggest that COX2/PGE_2_/EP4 is an important axis contributing to the adaptive response of the cells, enabling barrier restoration subsequent to initial disruption caused by DES.

### 3.6. Ascorbic Acid Prevents the Acute Barrier Permeability Induced by DES in HUVECs

**Figure 6:**
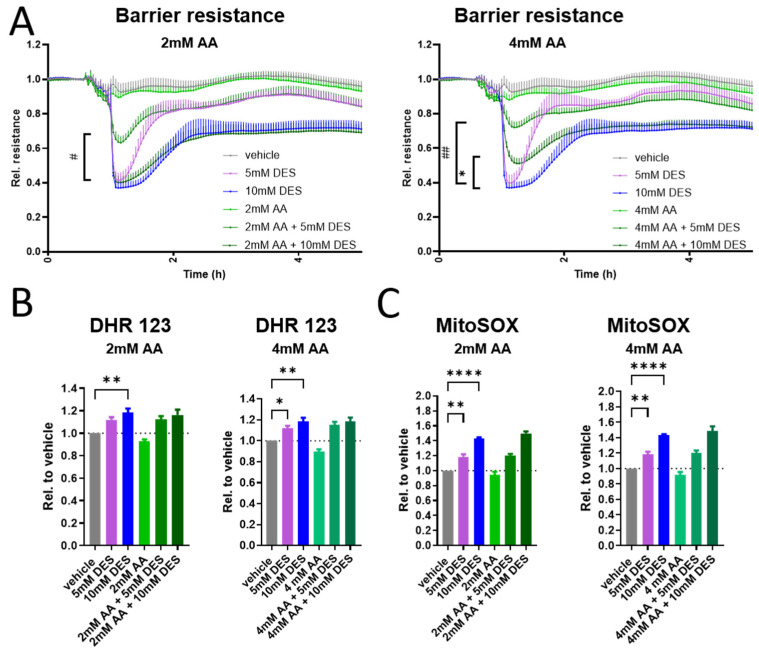
Ascorbic acid prevention of initial barrier drop induced by DES in HUVECs. (**A**) Resistance of HUVECs monolayer treated with ascorbic acid at demonstrated concentrations 15 min prior to stimulation with DES at indicated concentrations (n = 4). (**B**) DHR 123 geometric mean of fluorescence intensity in HUVECs stimulated with DES at indicated concentrations for 1 h with/without 15 min of ascorbic acid pre-treatment (n = 3). (**C**) MitoSOX geometric mean of fluorescence intensity in HUVECs stimulated with DES at indicated concentrations for 1 h with/without 15 min of ascorbic acid pre-treatment (n = 3). Data are presented as mean and SEM, with statistical significance determined using two-way ANOVA for repeated measures followed by Tukey’s post hoc test (**A**) or one-way ANOVA for repeated measures followed by Tukey’s post hoc test (**B**,**C**). For (**A**), * refers to comparison between 10 mM DES and AA + 10 mM DES, while # refers to comparison between 5 mM DES and AA + 5 mM DES. *, # *p* < 0.05; **, ## *p* < 0.01; **** *p* < 0.0001. AA denotes ascorbic acid.

In an attempt to inhibit the initial barrier drop caused by DES, we pre-treated the cells with ascorbic acid as a potent antioxidant [43]. Indeed, ascorbic acid pre-treatment reduced the initial barrier drop caused by DES, without having a barrier-enhancing effect alone on the cells (Figure 6A). Importantly, treating the cells with both ascorbic acid and DES simultaneously did not show a similar barrier response (Supplementary Figure S5A), suggesting that pre-treatment is a pre-requisite for this ameliorating effect and excluding the possibility that the amelioration is caused by an interaction between DES and ascorbic acid in cell culture medium.

To investigate whether ROS is directly involved in this barrier response, we repeated our ROS measurements at 1 h with/without ascorbic acid pre-treatment. Interestingly, ascorbic acid at the tested concentrations did not inhibit the increase in ROS production induced by DES in either DHR 123 or MitoSOX assays (Figure 6B,C), while reducing basal ROS production at matched times (Supplementary Figure S5B,C), suggesting that the protective effect of ascorbic acid might be mediated via other mechanisms that are not exclusively related to direct ROS scavenging. Collectively, our data indicate that ascorbic acid effectively prevents the barrier drop induced by DES, a response that is only reproducible if ascorbic acid was added prior to DES.

## 4. Discussion

In this study, we show that elevated intracellular succinate concentrations alter endothelial mitochondrial function as indicated by reduced activity in an MTS assay, elevated cellular ROS and mitochondrial superoxide production and a reduction in the mitochondrial membrane potential. A Seahorse mito-stress test corroborated these findings. While there were mixed pro-apoptotic and pro-survival signals in the cells at the gene level, this was not evident at the protein level and the cells rather had reduced caspase 3/7 activity in response to DES at a later time point. Additionally, there was no indication of cellular apoptosis in annexin V/propidium iodide staining. Meanwhile, DES stimulation triggered an acute concentration-dependent drop in endothelial barrier function. During prolonged stimulation, activation of the cells presented as a restoration of barrier integrity and cellular sprouting, which was concurrent with restoration of the mitochondrial membrane potential, albeit with continued ROS production. Furthermore, increased COX-2 protein expression and the release of PGE_2_ were observed. Inhibition of COX-2 and blocking EP4, but not EP2, receptors hampered the barrier recovery, suggesting a role of COX-2/PGE_2_/EP4 signalling in barrier restoration. Interestingly, pre-treatment of HUVECs with ascorbic acid inhibited the initial barrier permeability triggered by DES, independent from ROS inhibition.

In this study, we used DES in a mM concentration range. The rationale for using these relatively high concentrations originates from prior studies reporting that succinate concentrations rise drastically in pathological conditions such as ischemia–reperfusion injury and inflammation [18,44,45], with estimates suggesting local tissue concentrations in the mM range in cancer [27,28]. Furthermore, higher concentrations of stimuli (in our case DES) are often necessary to trigger robust and measurable cellular responses in vitro. This reflects the need for in vitro–in vivo scaling, where the dynamics of metabolite transport, clearance and tissue distribution cannot be fully replicated in a cell culture system [46]. In our study, lower DES concentrations did not elicit statistically significant changes in endothelial barrier resistance (measured in ECIS) or metabolic activity (assessed by MTS assay), although we cannot exclude that certain cellular functions could still be regulated by lower concentrations.

In our experiments, stimulation of HUVECs with DES triggered a gradual increase in cellular ROS and mitochondrial superoxide production. A strongly imposed mechanism by which succinate could increase ROS production is via reverse electron transfer (RET). For instance, in macrophages, LPS stimulates succinate-driven backward electron flow to complex I inducing the production of superoxide [32]. Concurrently, DES induced a reduction in mitochondrial membrane potential and ATP production, which could be caused by reduced activity of the electron transport chain in the cells. Interestingly, there was no significant change in ECAR, which is a proxy for cellular glycolysis, suggesting that the cells do not engage in further glycolysis as an alternative pathway, in this case of altered mitochondrial function. In agreement with this, it was previously proposed that glycolysis in endothelial cells does not function as a compensatory mechanism for reduced respiratory ATP supply during hypoxia [33], reinforced by the observation that lactate production during hypoxia is not significantly increased.

An increased ratio of BAX/BCL2L1 gene expression suggested initiation of an intrinsic apoptotic pathway in HUVECs in response to DES. The BCL-2 family is one of the most important regulators of intrinsic cellular apoptosis [47]. This family includes both pro-apoptotic members such as BAX and anti-apoptotic members such BCL-XL. Eventually, cellular fate is more dependent on the balance between pro- and anti-apoptotic signals than on the absolute quantity of the individual factor alone [48]. In the intrinsic apoptotic pathway, activation of caspase 3/7 occurs in apoptotic cells subsequent to mitochondrial membrane permeabilization and cytochrome-c release [49]. Interestingly, our data showed a reduction in caspase 3/7 activity in DES-treated cells independent of the cell numbers (measured as protein quantity), suggesting that the cells did not commit to the apoptotic pathway. While oxidative stress can be an inducer of intrinsic apoptosis [50], we do not have enough evidence that the elevated mitochondrial ROS production and the reduction in the mitochondrial membrane potential observed in our cells after treatment with DES culminated in cell apoptosis. Upregulation of VEGF gene expression substantiated the assumption that HUVECs’ survival and repair mechanisms are concurrently induced.

The response to DES in our ECIS experiments, which was replicated in the permeability assay, was rapid as we observed a substantial drop in HUVEC monolayer resistance within minutes post stimulation. Prompt cellular response to DES was previously demonstrated as DES increased carotid body (peripheral chemoreceptor) chemo-afferent activity, reaching a steady state within 3 min, and was rapidly reversible [51]. In our study, the reduction in HUVEC barrier function by succinate was paralleled by morphological changes in the cells presenting as cell shrinkage and an increase in F-actin proposing cytoskeleton remodelling. Nevertheless, the involvement of other mechanisms, such as the disruption or re-localization of key endothelial junction proteins, cannot be excluded and requires further investigation [52]. Recently, succinate accumulation in endothelial cells was linked to glycocalyx degradation in traumatic hemorrhage via membrane reorganization [53]. While a role of oxidative stress in endothelial permeability has been previously described [54,55], several lines of evidence suggest that ROS production by DES was not the primary cause of barrier permeability in our cell model. First, the dynamics of barrier drop and ROS production in the initial phase varied, as maximum barrier permeability was around 15 min post treatment, while maximum ROS production was observed at 1 h. Second, ROS production in the cells was sustained during barrier recovery. Third, while ascorbic acid ameliorated the barrier permeability, it was incapable of reducing the increase in ROS production induced by DES. Therefore, we propose that the small but statistically significant amount of ROS produced in our cells upon stimulation with DES might be required to maintain certain aspects of cellular signalling. Supporting this hypothesis, it was previously shown that endothelial cells exposed to chronic hypoxia accumulate succinate, which drives mitochondrial ROS production. Importantly, the authors highlighted that this increase in ROS was not excessive and was not associated with a reduction in cellular viability. Therefore, it might act as a regulator of cellular adaptation to hypoxia [56].

Notably, treatment with DES acutely reduced phosphorylation of ERK1/2 in comparison to baseline conditions. The role of ERK1/2 in regulating endothelial barrier function has been previously studied and yielded contradicting results [37,57,58,59]. In our study, the inhibition of ERK1/2 phosphorylation by U0126 enhanced the speed of barrier recovery following DES-induced barrier disruption. However, this response was not sustained as prolonged inhibition resulted in the deterioration of barrier integrity. These data emphasize that different molecular mechanisms might be involved at various stages of barrier response and that the balance between mechanisms driving permeability and those stabilizing the barrier function dictates the final response. Whether the activation of other intracellular signalling pathways, such as the modulation of intracellular calcium levels, might play a role in DES-induced barrier disruption requires further investigation. In contrast to DES, extracellular succinate is reported to induce ERK1/2 activation in several cell types in a SUCNR1-dependent manner, modulating distinct and even contradicting cellular responses such as apoptosis [60,61], activation [62] and angiogenesis [28].

After overnight stimulation with DES and concurrent with the restoration of the cellular barrier, increased expression of COX-2 was observed in our cell model and was associated with increased PGE_2_ release. While COX-2 is constitutively expressed in endothelial cells, its expression is upregulated by numerous factors, such as shear stress [63], hypoxia [64] and cytokines like TNF and IL-1 [65]. Of interest is that a previous study demonstrated that cytoskeleton-perturbing atherogenic and inflammatory stimuli induce COX-2 expression in endothelial cells, which was considered to be a vasoprotective mechanism [66]. In addition to these factors, oxidative stress seems to play an important role in the regulation of COX-2 expression and prostanoid profile in endothelial cells. As an example, human aortic endothelial cells cultured in high glucose increase their expression of COX-2 and alter their prostanoid profile in parallel to increased oxidative stress [67]. Similarly, in human monocytes activated with LPS, elevated COX-2 expression and PGE_2_ release were caused by oxidative stress [68]. While in our cell model we demonstrate increased oxidative stress and increased COX-2 expression in response to DES, a possible direct link between both responses still needs to be addressed. Supporting the protective role of COX-2, a previous study proposed that the elevation of COX-2 expression in patients with chronic obstructive pulmonary disease and in endothelial cells treated with cigarette smoke extract is a protective mechanism against endothelial cell apoptosis [69]. In our setting, COX-2 increase was associated with increased production of PGE_2_, which is known to have a protective role for endothelial cells, particularly under conditions in which the endothelial barrier is compromised [70]. Our data also suggest that barrier restoration is mediated, at least partly, via EP4 since the blockade of this receptor using ONO-AE3-208 compromised the barrier recovery. The role of this receptor in mediating PGE_2_ and PGD_2_ barrier-enhancing function was shown in previous studies from our group [71,72].

While cellular adaptation after prolonged DES stimulation was observed in our study, this does not necessarily reflect the situation in vivo. It is plausible that endothelial cells chronically exposed to elevated succinate levels (e.g., in tumours or inflamed tissues) may sustain a basal state of dysfunction rather than complete recovery. This could manifest as persistent barrier permeability, chronic oxidative stress, or metabolic reprogramming, potentially contributing to disease progression. To address this hypothesis, we plan to extend our findings to in vivo models, particularly for cardiovascular diseases.

Notably, in our cell model, DES did not cause changes in HIF-1α protein expression at the used concentrations. Similarly, no stabilization of HIF-1α was observed in cultured human embryonic kidney (HEK293), hepatocellular carcinoma (Hep3B) and neuroblastoma (Kelly) cells and fibroblasts (CRL-2086) upon treatment with succinate diethyl or dimethyl ester [73]. In contrast, succinate dehydrogenase inhibition and subsequent increased succinate concentrations in HEK cells were shown to stabilize HIF-1α under normoxia, a phenomenon commonly referred to as pseudo-hypoxia and that is suggested to play a role in oncogenesis [31]. It is to be noted that the afore-mentioned study used dimethyl succinate at a higher concentration than the concentration used in our study and for longer time period. Hence, HIF-1α stabilization by succinate seemingly depends on many biological and experimental factors.

Ascorbic acid is an essential micronutrient that functions as a potent antioxidant and a critical cofactor for enzymes that are involved in multiple cellular activities [74]. Our data demonstrate that pre-treatment with ascorbic acid reduced the acute drop in HUVECs barrier caused by DES, suggesting a barrier-protective effect. In line with our findings, it was previously demonstrated that ascorbic acid enhances alveolar epithelial cell barrier integrity and prevents cytoskeleton remodelling upon exposure to proinflammatory stimuli [75]. Additionally, a role of ascorbic acid in enhancing the barrier of cultured bovine aortic and venous endothelial cells, and HUVECs was demonstrated [76]. Further, ascorbate inhibited VEGF-induced vascular permeability [77], and cell-free hemoglobin-induced permeability in HUVECs [78]. Similarly, ascorbic acid inhibited oxidized low-density lipoprotein [79] and thrombin-mediated permeability in EA.hy926 cells [80]. At the used concentrations and pre-treatment duration, ascorbic acid failed to inhibit the ROS production induced by DES, eliminating the possibility that the barrier-protective effect is a direct antioxidant activity of ascorbic acid. Nevertheless, the possibility that ascorbate-mediated regulation of dioxygenases in the cytoplasm or another mechanism is contributing to this response is to be addressed. Notably, the concentrations of ascorbic acid used in our assays are within the physiological ascorbate concentrations, i.e., up to 5 mM in most tissues and to 10 mM in particular organs such as neurons [81].

In conclusion, our data demonstrate for the first time that succinate elevation inside endothelial cells results in alterations in mitochondrial function and barrier permeability. This initial response is seemingly counterbalanced by increased VEGF expression and prostaglandin production from the cells, eventually culminating in gradual restoration of cellular barrier, sprouting and recovery of mitochondrial membrane potential, while sustaining a state of elevated ROS production.

## Data Availability

All data generated in the current study are available upon reasonable request from the corresponding author.

## Supplementary Materials

The following supporting information can be downloaded at https://www.mdpi.com/article/10.3390/antiox13121579/s1. Figure S1: Effect of low DES concentrations on HUVEC metabolic activity and barrier function; Figure S2: Corresponding ECAR of HUVECs recorded in Seahorse mito-stress test; Figure S3: Effect of DES stimulation on HUVEC permeability; Figure S4: Regulation of VE-cadherin protein expression and HIF-1α gene expression by DES and effect of ERK1/2 inhibition on DES-induced change in barrier resistance; Figure S5: Effect of ascorbic acid co-treatment on DES-induced barrier permeability and on basal ROS production.
