# Supplementary material for: Succinate Regulates Endothelial Mitochondrial Function and Barrier Integrity
Source: Antioxidants (Basel). 2024 Dec 21;13(12):1579. doi: 10.3390/antiox13121579 (PMC11673088; doi:10.3390/antiox13121579)
Supplement: Supplementary file 1 [file antioxidants-13-01579-s001.zip › antioxidants-3345642-supplementary.pdf]

# Succinate Regulates Endothelial Mitochondrial Function and Barrier Integrity

Supplementary Figures

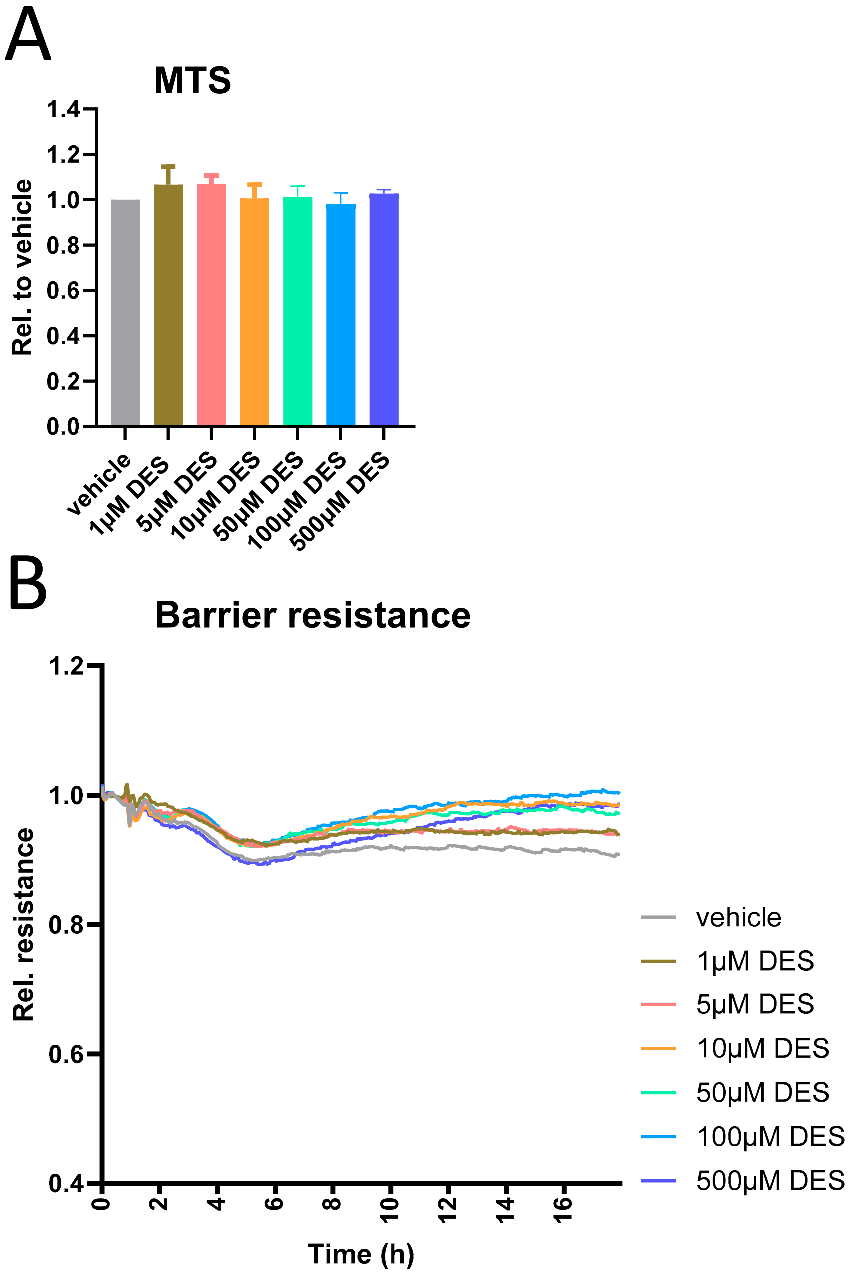


**Supplementary Figure S1.** Effect of low DES concentrations on HUVECs metabolic activity and barrier function. (**A**) MTS assay of HUVECs stimulated with DES at indicated concentrations for 16 h (n = 3). (**B**) Resistance of HUVECs monolayer stimulated with DES at indicated concentrations (n = 3). (**A**) is presented as mean and SEM. (**B**) is presented as mean for better visualization.

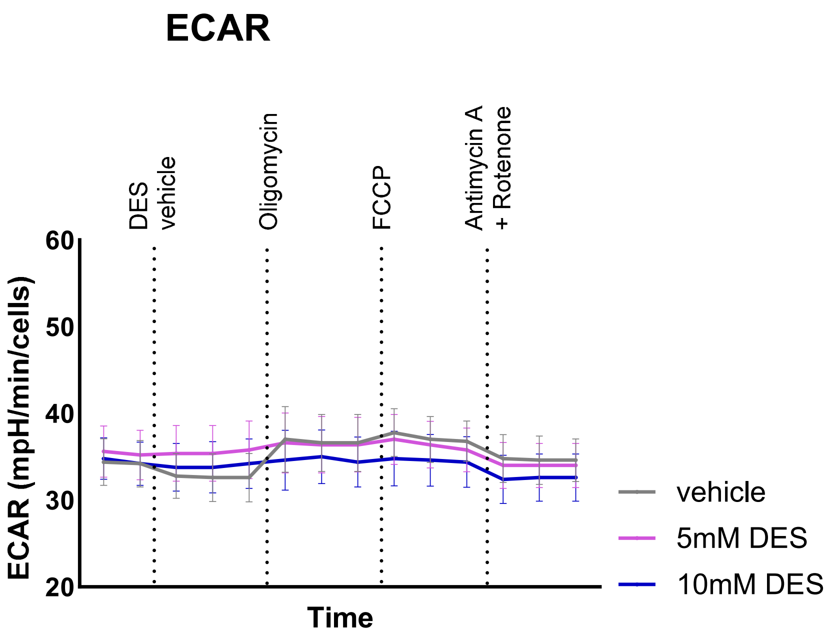


**Supplementary Figure S2.** Corresponding ECAR of HUVECs recorded in Seahorse mito-stress test. Data are presented as mean and SEM (n = 5).

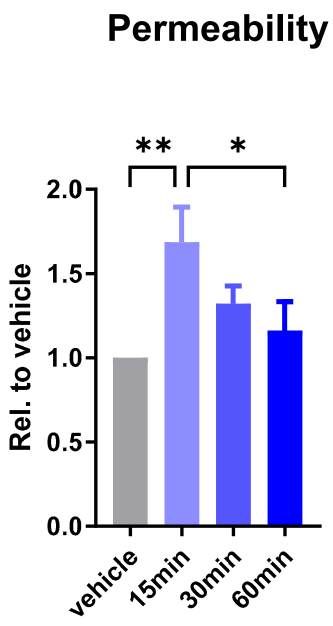


**Supplementary Figure S3.** Effect of DES stimulation on HUVECs permeability. FITC-dextran permeability assay of HUVECs stimulated either with vehicle or 10 mM DES for indicated time points (n = 4). Data are presented as mean and SEM, with statistical significance determined using one-way ANOVA for repeated measurements followed by Tukey's post hoc test. * $p < 0.05$; ** $p < 0.01$.

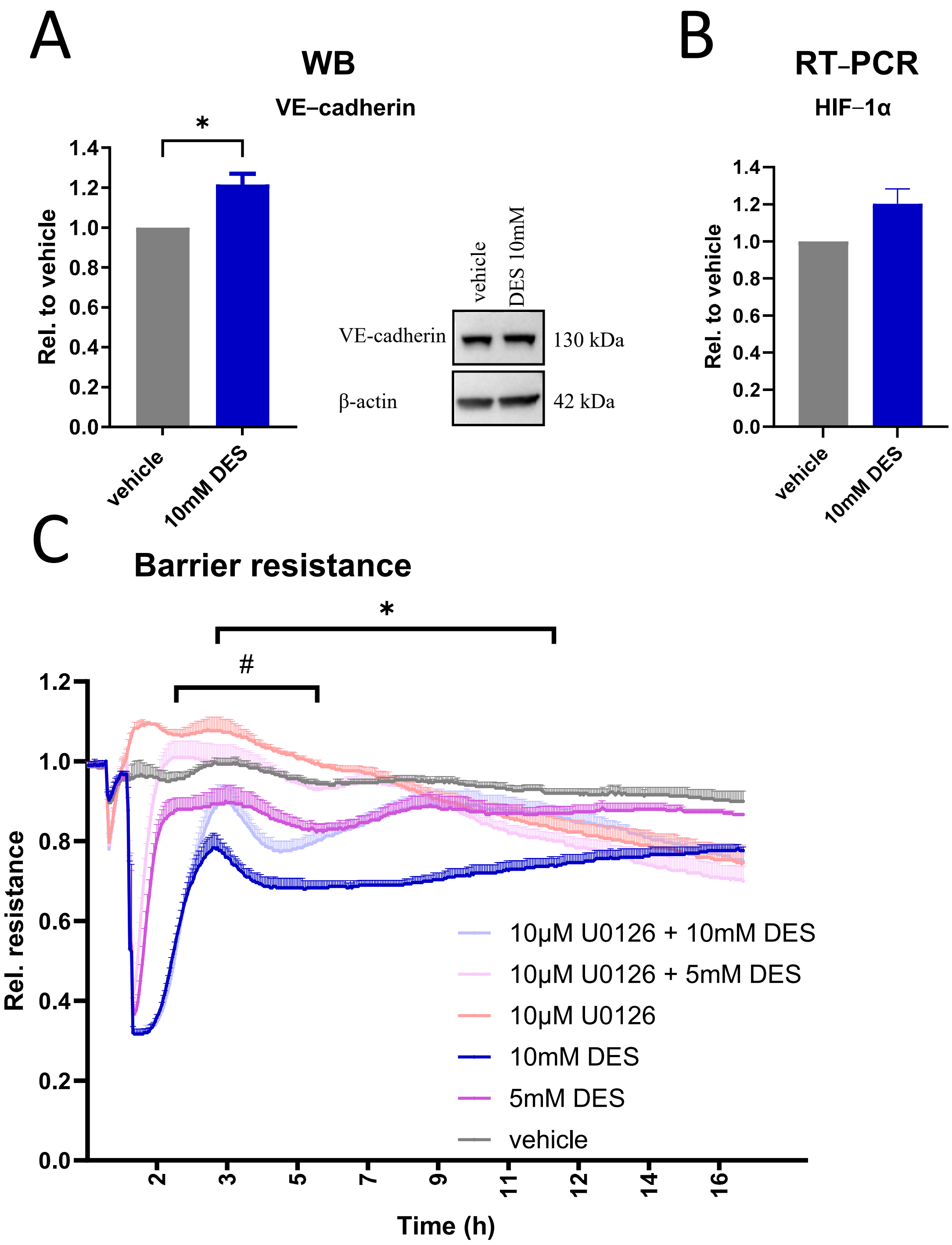


**Supplementary Figure S4.** Regulation of VE-cadherin protein expression and HIF-1*α* gene expression by DES and effect of ERK1/2 inhibition on DES-induced change in barrier resistance. (**A**) Western blot of VE-cadherin in HUVECs after 16 h of stimulation with 10 mM DES (n = 4). (**B**) RT-PCR of HIF-1*α* mRNA expression in HUVECs stimulated with 10 mM DES for 4 h (n = 6). (**C**) Resistance of HUVECs monolayer treated with 10 µM U0126 followed by stimulation with indicated DES concentrations (n = 3). Data are presented as mean and SEM, with statistical significance determined using paired *t*-test (**A**,**B**) or two-way ANOVA for repeated measures followed by Tukey's post hoc test (**C**). For (**C**), * refers to comparison between 10 mM DES and U0126 + 10 mM DES, while # refers to comparison between 5 mM DES and U0126 + 5 mM DES. *, # $p < 0.05$.

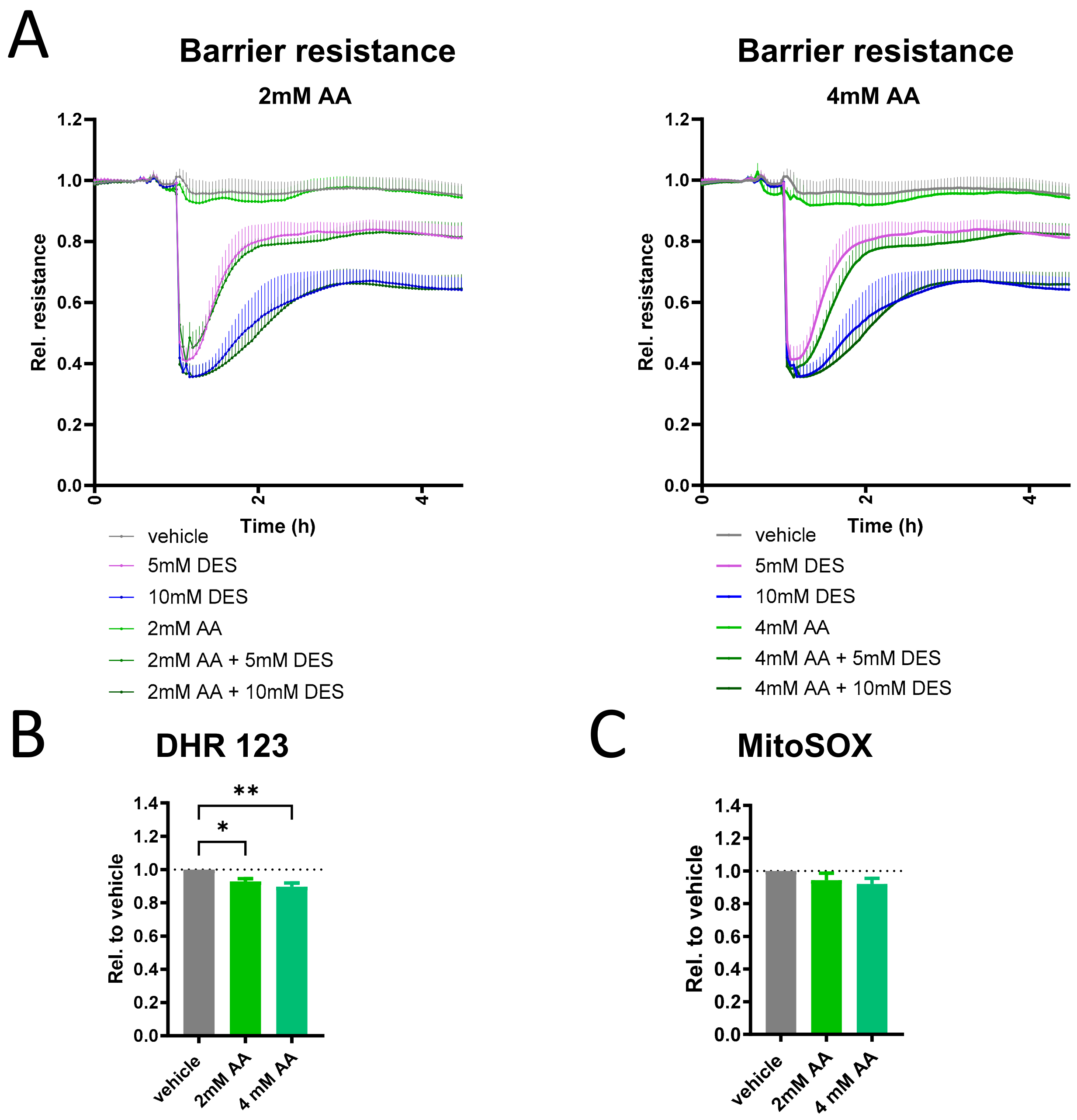


**Supplementary Figure S5.** Effect of ascorbic acid co-treatment on DES-induced barrier permeability and on basal ROS production. (**A**) Resistance of HUVECs monolayer treated with ascorbic acid at demonstrated concentrations concurrent with stimulation with indicated DES concentrations (n = 4). (**B**) DHR 123 geometric mean of fluorescence intensity in HUVECs treated with ascorbic acid for 75 min (n = 3). (**C**) MitoSOX geometric mean of fluorescence intensity in HUVECs treated with ascorbic acid for 75 min (n = 3). Data are presented as mean and SEM, with statistical significance determined using one-way ANOVA for repeated measures followed by Tukey's post hoc test (**B**,**C**). * $p < 0.05$; ** $p < 0.01$. AA refers to ascorbic acid.
